# An Exercise Prescription for Patients with Stroke and Sarcopenia Based on the Modified Delphi Study

**DOI:** 10.3390/life14030332

**Published:** 2024-03-01

**Authors:** Jae Wan Yoo, Geun-Young Park, Hae-Yeon Park, Yeun Jie Yoo, Mi-Jeong Yoon, SeungYup Song, Kyung Hyun Park, Hooman Lee, Sangui Choi, Sun Im, Seong Hoon Lim

**Affiliations:** 1 Department of Rehabilitation Medicine, Graduate School of Medicine, The Catholic University of Korea, Seoul 06591, Republic of Korea; ainshu@gmail.com; 2 Department of Rehabilitation Medicine, RHIN Hospital, Yongin-si 16864, Republic of Korea; 3 Department of Rehabilitation Medicine, Bucheon St. Mary’s Hospital, College of Medicine, The Catholic University of Korea, Seoul 06591, Republic of Korea; rootpmr@catholic.ac.kr (G.-Y.P.); hy2park@naver.com (H.-Y.P.); footballkid@naver.com (S.S.); 4 Department of Rehabilitation Medicine, St. Vincent’s Hospital, College of Medicine, The Catholic University of Korea, Seoul 06591, Republic of Korea; yeunjie@catholic.ac.kr (Y.J.Y.); allogen@naver.com (M.-J.Y.); 5 Department of Rehabilitation Medicine, Seoul St. Mary’s Hospital, College of Medicine, The Catholic University of Korea, Seoul 06591, Republic of Korea; pkh1338@naver.com; 6 The Research and Development Center, Exosystems, Seongnam-si 13449, Republic of Korea; hoomanlee@exosystems.io (H.L.);

**Keywords:** stroke, sarcopenia, Delphi, exercise, hemiplegia

## Abstract

Background: We aimed to develop a consensus on the need for and priorities of exercise to treat preexisting sarcopenia with hemiplegic stroke. Methods: A modified three-round Delphi study was conducted. The panelists responded to the questionnaire on a 7-point Likert scale. Responses were returned with descriptive statistics in the next round. Consensus was defined as >75% agreement (score of 5–7) with a median > 5. The percentage of strong agreement (score of 6–7) and Kendall’s coefficient of concordance were calculated to demonstrate a more refined interpretation of the consensus. Results: Fifteen panelists contributed to all rounds. The need for exercise was demonstrated. The consensus was reached on 53 of 58 items in the first round and all items in the second and final rounds. The percentage of strong agreement was high for all but eight items. Conclusions: This study is the first Delphi study to investigate the need for and priorities of exercise for treating preexisting sarcopenia in stroke hemiplegia. We present a standard recommendation including 57 priorities and a strong recommendation including 49 priorities. The eight items that were excluded reflected factors that are less important to hemiplegic patients with poor balance, cognitive decline, or mental vulnerability.

## 1. Introduction

Sarcopenia is a progressive and systemic disease characterized by decreased muscle strength and muscle mass. It is associated with functional decline resulting in fragility, falls, and disability [1,2,3]. The current diagnostic criteria for sarcopenia are based on the revised definitions from the 2018 European Working Group on Sarcopenia in Older People (EWGSOP) [2] and the 2019 Asian Working Group for Sarcopenia (AWGS) [3]. Sarcopenia is diagnosed when low muscle strength and low muscle mass quantity/quality are confirmed according to EWGSOP and when loss of muscle mass plus low muscle strength and/or low physical performance are confirmed according to AWGS. Severe sarcopenia is considered when all three conditions (low muscle strength, low muscle mass, and low physical performance) are met in both groups. Several measurement methods are used to check the criteria. For example, hand grip strength is used for muscle strength. Appendicular skeletal muscle mass, bioimpedance, and ultrasound are used for muscle mass. Gait speed and short physical performance battery are used for physical performance. The prevalence of sarcopenia is about 29% in the elderly and up to 33% in hospitalized patients [4,5]. Sarcopenia is divided into primary origin, which is age-related, and secondary origin, which is disease-related, according to etiology. Previous studies have revealed that secondary sarcopenia is related to stroke, cardiovascular disease, inflammatory disease, endocrine disorders, disuse, and malnutrition [6,7,8]. Because sarcopenia harms the quality of life of the elderly and increases the risk of hospitalization, preemptive prevention and treatment are crucial. There are a variety of treatments including exercise and dietary interventions to improve sarcopenia [9]. Especially, exercises such as resistance training and endurance training have been demonstrated to be effective in treating sarcopenia [10,11].

Stroke is a major cause of morbidity and the second leading cause of mortality [12]. Indeed, sarcopenia has been reported in 53% of stroke patients, which is higher than that in the general elderly population [13,14]. Recent studies on the relationship between sarcopenia and stroke have been conducted [8,15,16,17,18,19,20,21,22]. Some authors have investigated the negative effect of pre-existing sarcopenia on stroke [16,17,19]. Others have studied distinct features of stroke-induced sarcopenia, which is different from that observed with aging. Stroke-induced sarcopenia results in a rapid decline in muscle mass and structural muscle alterations, accompanied by denervation, remodeling, atrophy, spasticity, inflammation, and malnutrition [8,18,19]. Studies have confirmed that sarcopenia may delay functional recovery from a stroke, regardless of whether the sarcopenia developed before or after the stroke. Therefore, for patients with stroke and sarcopenia, it is important to treat the affected limb of the stroke and also to treat sarcopenia in all limbs, including the non-affected limbs. Several studies on exercise as a treatment for sarcopenia using the Delphi method have been performed [23,24]. Research on exercise in other diseases, such as musculoskeletal or internal medicine, has also been conducted [25,26,27,28,29]. However, studies on exercise targeting patients with both stroke and preexisting sarcopenia are rare.

Stroke patients have limitations in terms of exercise, including loss of balance. Exercise to treat sarcopenia in stroke patients needs to be discussed separately from that of non-stroke patients. Therefore, this study aims to develop exercise priorities to treat sarcopenia when hemiplegic stroke occurs in patients with sarcopenia, using the Delphi process [30,31,32,33].

## 2. Materials and Methods

### 2.1. Study Design

The Delphi study is a methodology of iterative surveys used to develop consensus in a given field requiring experts’ consensus. In the process, panelists respond to a given questionnaire and respond repeatedly through feedback in subsequent surveys. The composition of panelists may vary, including researchers, stakeholders, or patients to collect diverse opinions. The number of surveys or rounds is not set, but usually 2 to 3 rounds are conducted and then consensus is reached between the panelists according to predefined criteria. The modified Delphi process, in which panelists allow the questionnaire to be modified by adding new items or modifying previous items, can be also used.

We conducted a modified Delphi study according to the guidance on conducting and reporting Delphi studies [33]. Three Delphi rounds were used to collect the judgments from the experts on given themes.

### 2.2. Participants

The Delphi panelists were recruited by using purposive sampling. To ensure diverse perspectives, we intended to recruit participants from various professions (physicians, therapists, and nurses). We set separate eligibility criteria for each group to secure expertise while pursuing diversity. The criteria for physicians were to be clinical or academic experts specializing in areas related to exercise such as rehabilitation medicine, orthopedics, neurology, or neurosurgery and to have more than five years of professional experience with at least one article in a peer-reviewed journal in the fields of exercise, sarcopenia, or stroke. Physical or occupational therapists were eligible when participants belonged to hospitals with a certified license and had experience in exercise therapy for at least 3 years. Since systematic implementation and long-term treatment experience are important in exercise therapy, work experience at a hospital rather than a small clinic was included as a recruitment condition. The criteria for nurses were to be a registered nurse; have at least 3 years of clinical or research experience; and be involved in a ward or a laboratory of rehabilitation medicine, orthopedics, neurology, or neurosurgery. Contact information of eligible participants was verified through a PubMed search, affiliated hospitals’ websites, public notice, or referrals from peers. All eligible individuals were contacted via e-mail to confirm their consent to participate, and the Delphi survey was conducted only for those who agreed. Characteristics such as age, gender, or duration of experience were collected only from those who consented. This study was approved by the Ethics Committee of the Catholic University of Korea (HC23QNDE0007).

### 2.3. Sample Size

The optimal panel size has not been established. The number of panelists in several Delphi studies varied from as few as a dozen to as many as several hundred participants [31,32,34]. We planned to recruit 5–7 individuals per group and 15–21 individuals overall.

### 2.4. Delphi Process

**Figure 1:**
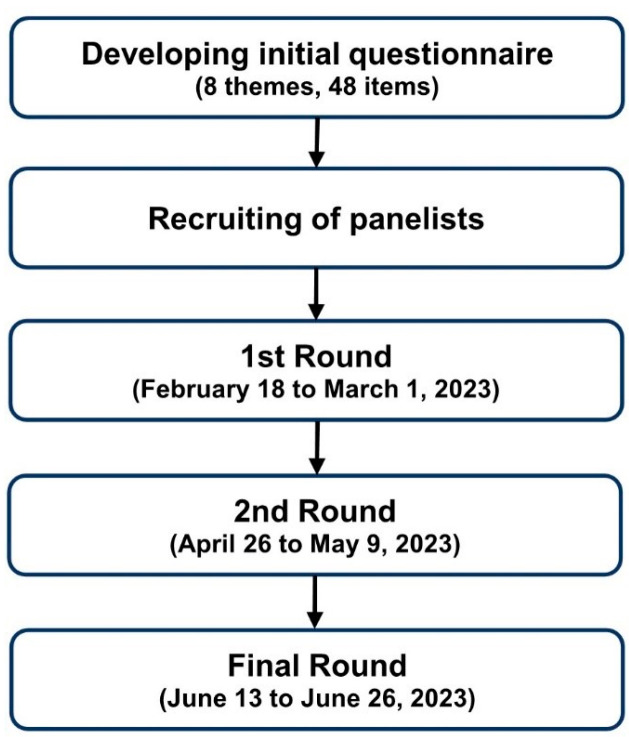
Flow diagram of the Delphi study.

Three survey rounds were conducted via e-mail with the attached questionnaire or web-based survey (Google Form) from February to June 2023. The questionnaire consisted of 58 items in 8 themes, and detailed questions were shown in Section 3 Results. Each round lasted 2 weeks, after which one or two reminder e-mails were sent to non-respondents. The schematic study flow diagram is shown in Figure 1. 

### 2.5. Round 1

Three authors (Yoo, J.W.; Im, S.; and Lim, S.H.), who were all specialists in rehabilitation medicine, participated in developing the questionnaire. We proceeded in two directions. One was to identify underlying aspects to be considered in exercise prescription through a literature review, and the other was to summarize the functional limitations of patients with sarcopenia or stroke hemiplegia, such as loss of balance, muscle weakness, or cognitive decline. We conducted a literature search in PubMed using the term “exercise”, “Delphi”, “consensus”, and “recommendation”. Approximately 900 papers, including systematic reviews, recent studies, and clinical guidelines, were retrieved. We briefly reviewed the papers based on whether they were well categorized and detailed on exercise components, and studies for rare diseases were excluded for generality. Several high-quality papers on exercise in various diseases were selected [23,24,25,26,27,28,29]. Among them, we adopted the results of a well-structured study [28]. Then, to reflect the limitations of stroke or sarcopenia patients, several items were added including the need for exercise equipment in patients with impaired function, the need for additional methods such as stretching or flexibility exercises, and a detailed description of the amount of exercise. Two items (change of exercise position and change of range of motion) were modified for clarity of meaning. Finally, the initial questionnaire with 58 items in 8 themes was complete, including an item about the need for an exercise program to treat sarcopenic patients with stroke hemiplegia. A free comment space was provided for additional opinions. A panel of experts was asked to rate their agreement with the statements in the questionnaire on a 7-point Likert scale (1, strongly disagree; 2, disagree; 3, somewhat disagree; 4, neutral; 5, somewhat agree; 6, agree; 7, strongly agree). 

### 2.6. Round 2

The second questionnaire was distributed to respondents who completed the first questionnaire. The first-round survey results were used along with their previous scores and descriptive statistics (median, interquartile range (IQR), and percentage of agreement). Two additional open-opinion responses were collected. One was about exercise modification to the patient—that is, the duplicate of item 1 of Theme 1. The other was about home exercise. Since this study targeted hemiplegic stroke patients with sarcopenia, exercise should be performed under the supervision of an expert. Self-exercise at home, which cannot be monitored, was considered inappropriate for this study. Therefore, we decided not to include duplicate or inappropriate questions in the second round. As a result, the items in the second round remained the same as those in the first round. The panelists were asked to re-rate their agreement with a statement using the same 7-point Likert scale. A free comment space was provided as in the first survey.

### 2.7. Round 3

The final questionnaire was distributed to those who responded to the first and second questionnaires. The panelists were asked to re-rate their agreement in the third survey with feedback from the previous round as in the second round. As no new open comments were provided in the previous round, the overall items remained the same.

### 2.8. Statistical Analysis

The statistical values for each round, such as median, IQR, and percentage of agreement (score of 5–7), were calculated and used as feedback for the next round. Consensus was defined as more than 75% agreement (score of 5–7) with a median > 5. To determine the trend in the agreement through rounds, all items were retained regardless of whether an agreement was reached. The percentage of strong agreement (score of 6–7) was calculated to refine the interpretation of the consensus. Kendall’s coefficient of concordance was used to evaluate inter-expert agreement across statements [35,36]. The exercise priorities to treat sarcopenia were recommended for patients with hemiplegic stroke based on the results of the final round. All statistical analyses were performed using SPSS software (ver. 28.0; SPSS Inc., Chicago, IL, USA).

## 3. Results

### 3.1. Participant Characteristics

Twenty one individuals were contacted and fifteen agreed to participate in the Delphi study. All participants responded to all three rounds of questionnaires (response rate = 100%). The characteristics of the participants in the Delphi study are shown in Table 1. Five physicians had an average of 20.2 years (14–26 years) of clinical experience. They were professors and clinicians at a total of three university hospitals, and each was involved in several positions in representative domestic medical societies or journals. Five therapists and five nurses had an average of 11.6 years (8–14 years) and 15.8 years (5–25 years) of working experience, respectively. They were recruited from one university hospital and one rehabilitation hospital. All of them worked in rehabilitation wards or a rehabilitation-related laboratory of hospitals and took on leadership roles in their respective workplaces. The panelists were involved in a total of four hospitals (three university hospitals and one rehabilitation hospital). The detailed characteristics of the panelists and hospital information, including hospital type, size, and number of hospitalized patients, are provided in the Supplementary Material (Table S1).

### 3.2. Consensus

#### 3.2.1. Need for Exercise

The item regarding the need for exercise met the consensus definition in all three rounds. The overall median score was 6, with an IQR of 1.0, and the percentage of agreement (score of 5–7) was 100% for each round.

#### 3.2.2. Priority of Exercise

The questionnaire consisted of 57 items and eight themes (general concern, exercise dosage, exercise type, additional exercise, evaluation of exercise, progressive overload, when to overload, and how to overload). A consensus was reached on almost all items in the first round, except five (the need for an achievable challenge, collaboration with the patient, improvement of subjective symptoms in a general theme, need for flexibility exercise in an additional exercise theme, and the need for a complexity evaluation in the evaluation theme). Those five items had a median > 5, but the percentage of agreement was <75%. Those met the consensus criteria for the second and final rounds—that is, all 47 items met the consensus in the final round (Table 2).

### 3.3. Percentage of Strong Agreement

The percentage of strong agreement (score of 6–7) was introduced to interpret the results in more detail. The percentage of strong agreement was similarly high for most items, as was the percentage of agreement, with some mismatch. There were eight items with a percentage of strong agreement < 50% in the final round. Those were the need for collaboration with the patient (47% strong agreement, 93% agreement), the need to improve subjective symptoms (47% strong agreement, 87% agreement), the need for weight equipment such as ankle weights (27% strong agreement, 80% agreement), the need for elastic equipment such as a Thera band (27% strong agreement, 87% agreement), the need for flexibility exercises such as yoga (33% strong agreement, 80% agreement), the need for a complexity evaluation (40% strong agreement, 93% agreement), the need for progressive overload when symptoms decreased (33% strong agreement, 93% strong agreement), and the need for additional equipment for progressive overload (33% strong agreement, 100% agreement) (Table 3).

### 3.4. Kendall’s Coefficient of Concordance

The inter-expert agreement was significant (*p* < 0.05) for all rounds across all items and across items within themes (Table 4). All Kendall’s W, the strength of inter-expert agreement, ranged from 0.2 to 0.4, indicating fair agreement [37].

## 4. Discussion

Various treatments for sarcopenia have been recommended in previous studies [9,10,11,38]. Holistic approaches, including dietary and exercise interventions, could be effective to limit the occurrence and severity of sarcopenia [9]. A balanced diet with appropriate calorie intake, high in protein, Ca^2+^, Mg^2+^, and vitamin D, reduced the likelihood or progression of sarcopenia. Resistance exercise with moderate loads and volumes was thought ideal to treat sarcopenia. Moreover, the author recommended nutritional-resistance exercise intervention—that is, dietary and exercise interventions needed to be performed in combination, not alone, to mitigate sarcopenia. Other authors focused more on exercise as a treatment for sarcopenia. Strength training or resistance exercise with appropriate loads during a well-organized, individualized, periodized, and monitored program, could be a promising treatment for sarcopenia of elderly population by improving muscle strength, power, hypertrophy, and endurance [10,38]. Several types of resistance exercise such as suspension-based resistance training (S-RT), cluster set resistance training, high-speed resistance training, and low-load power-based training were introduced in one study [10]. However, the need for future research was also emphasized for practical recommendations of S-RT. Another study revealed that a balanced program of both strength and endurance exercises on a regular schedule, at least 3 days a week, was the effective strategy to improve sarcopenia in older adults. The author also pointed out that it was not yet clear what exercise was recommended for elderly patients with physical limitations [11].

As mentioned above, several studies have investigated exercises for sarcopenic patients, but research on exercise for patients with both stroke hemiplegia and preexisting sarcopenia is rare. This Delphi study is the first to recommend exercise priorities for patients with preexisting sarcopenia in stroke hemiplegia.

We recruited a panel of fifteen experts, consisting of five participants from each of three subgroups. To ensure diversity and expertise, we set reasonable criteria for each group. All panelists were required to have sufficient clinical experience (more than five years for physicians and more than three years for therapists and nurses). In addition, they needed to have a certain level of academic or theoretical background (at least one article of a peer-reviewed journal for physicians and a certified license for the others) and the affiliation of all participants was limited to four departments (rehabilitation medicine, orthopedics, neurology, and neurosurgery) related to exercise, sarcopenia, or stroke. As a result, the panelists comprised individuals with abundant clinical experience and expertise. The overall average experience of the panelists was 15.9 years. All physicians held important positions in academic or clinical societies in Korea, and most therapist and nurses worked as team heads or team leaders. These qualified panelists provide reliability to this Delphi study. 

We used the percentage of agreement and the percentage of strong agreement to confirm exercise priorities and to refine them according to the degree of agreement. A standard recommendation was made by consensus definition—that is, the percentage of agreement was >75%. A strong recommendation was based on the criterion that the percentage of strong agreement was >50%. The cutoff value was lower than the standard version because the proportion of respondents decreased as the strength of agreement increased. We present the priorities consisting of 57 items for standard recommendation and 49 items for strong recommendation (Table 5). The eight excluded items reflect factors that are less important to hemiplegic patients with poor balance, cognitive decline, or mental vulnerability.

### 4.1. General Theme

We recommended 15 exercise priorities, including modification, simplicity, reality, achievable challenge, acceptability, proper time, adherence, collaboration with the patient, functional status and goals of the patient, the effect on neuromuscular performance, improvement of patient symptoms, patient monitoring, adaptation at the rehabilitation stage, patient education about exercise, and equipment availability. Two priorities, which are collaboration with the patient and improvement in patient symptoms, were excluded from the strong recommendation. Exercise for sarcopenic patients requires a multidimensional approach based on various priorities [20,21]. Considering the pathogenesis of hemiplegic stroke, the cognitive and mental status of these patients is often impaired to varying degrees. It is reasonable for exercise to focus on an objective function rather than subjective aspects of sarcopenic patients with hemiplegic stroke, and the results of our strong recommendation reflect this.

### 4.2. Dosage Theme

All six items investigated were included in the standard and strong recommendations, including frequency, intensity (of patient’s effort), load (of objective weight), repetitions per set, number of sets, and exercise duration. Sarcopenic patients with stroke hemiplegia often have various limitations, such as old age, poor muscle strength, poor physical condition, or underlying disease. It is important to adjust the exercise setting for each patient to reflect the priorities mentioned above.

### 4.3. Type Theme

Nine items were included as priorities in the standard version, including sequence, position, target muscle, range of movement, direction of resistance, use of equipment, use of weight equipment (e.g., ankle weights), use of elastic equipment (e.g., Thera band), and use of balancing equipment (e.g., wall or cane). Among them, the use of weight equipment and elastic equipment were excluded from the strong version. In general, the use of equipment (weight, elastic, or balancing devices) is recommended for patients with functional limitations to compensate for their function. Balancing devices need to be considered more important than others for hemiplegic patients who experience loss of balance due to low muscle strength on the affected side.

### 4.4. Additional Exercise Theme

We investigated exercise priorities by classifying them into various categories (aerobic, stretching, flexibility, and core muscle exercise) separately from extremity-strengthening exercises. All four items were recommended in the standard version, but flexibility exercise was not included in the strong recommendation. Priority or preference for an exercise depends on whether the exercise is necessary for the patient and whether the exercise can be performed by the patient. Patients with poor balance have difficulty performing flexibility exercises unless postural stability is achieved. For those, strengthening or aerobic exercise needs to be preceded by flexibility exercise.

### 4.5. Evaluation Theme

The exercise setting should be adjusted appropriately for each patient and each functional stage of the patient. We examined the priority needed for an exercise evaluation during adjustment of the exercise setting. All seven items were recommended in the standard version, including complexity, effort, compliance, pain during and after exercise, and fatigue during and after exercise. The strong recommendation was similar to the standard version except for complexity. The difficulty of the exercise does not matter concerning the functional or cognitive limitations of the patient. Rather, how well the patient adapts and performs is a more important factor to consider.

### 4.6. Overload Themes

We confirmed the need for progressive overload exercise. Then, we considered when and how overload should proceed. Six items were included in the standard recommendation for the “when to overload” theme; “when symptoms decrease” was excluded from the strong version. Nine items were recommended in the standard version for the “how to overload” theme, and eight items were included, excluding additional equipment, in the strong version (Table 5). In general, it is better to overload an exercise safely and reasonably when a patient can tolerate it or objectively when the goal has been reached. The subjective symptoms expressed by the patient may not exactly reflect the patient’s function due to cognitive or mental deficits, so they are not considered an important priority. The use of equipment may compensate for deficient functions during pre-overload exercise, but it could be considered dangerous to the patient during the overloading exercise. The items excluded from the strong versions of these themes were patient obstacles.

### 4.7. Study Limitations 

There were several limitations to be considered in this Delphi study. First, lack of generalizability needs to be considered. Our panelists were recruited from four hospitals including three university hospitals and one rehabilitation hospital in Korea. To address this limitation, we aimed for a variety of professions (physicians, therapists, and nurses) and included various majorities within each profession, shown in Table 1. Second, the number of panelists was relatively small. Although the optimal panel size has not been established [31,32,34], there is a need to conduct future research with more participants from a variety of countries to ensure generalizability. Third, we did not consider the recovery time between exercises. For patients with reduced physical function due to stroke or sarcopenia, it is important to consider recovery time for effective exercise. Fourth, our panelists did not include experts who specialize mainly in physical exercise prescription. Although rehabilitation medicine specialists, therapists, and nurses in rehabilitation wards have some degree of involvement in exercise prescription, the absence of experts in physical exercise prescription still serves as a weakness of this study. Finally, we did not adequately account for the difference among different subgroups of the panelists. In fact, most items reached consensus in the final round, and inter-expert agreement of entire panelists was confirmed using the Kendall’s coefficient. Nevertheless, it was possible that differences among subgroups were missed because the number of participants in each subgroup was limited. For more detailed analysis, follow-up research needs to secure a greater number of panelists in the subgroups. Despite its limitations, this study is the first on exercise priorities for pre-existing sarcopenia in stroke hemiplegia in one country and can serve as a reference guide worldwide.

## 5. Conclusions

This is the first Delphi study to investigate the need for and priorities of exercise to treat sarcopenia before stroke hemiplegia. We presented the two recommendations, which were the standard version including 57 priorities and the strong version including 49 priorities, which reflected the specific conditions of stroke hemiplegia, such as poor balance, cognitive decline, or mental vulnerability. These recommendations will be a useful guideline when considering exercise as a treatment for sarcopenia with stroke hemiplegia. Furthermore, the results could be meaningful for future research in the field of sarcopenia and stroke.

## Figures and Tables

**Table 1:** Characteristics of the Delphi panelists (N = 15).

	Participants, *n* (%)
Age, yrs, mean (range)	41.3 (28–59)
Female sex	8 (53.3)
Profession	
Physician—Physiatrist	3 (20.0)
Physician—Neurosurgeon	1 (6.7)
Physician—Neurologist	1 (6.7)
Therapist—Physical	3 (20.0)
Therapist—Occupational	2 (13.3)
Nurse—Clinical	4 (26.6)
Nurse—Research	1 (6.7)
Affiliation	
University hospital	10 (66.7)
Rehabilitation hospital	5 (33.3)
Experience period	
<10 yrs	2 (13.3)
10–19 yrs	8 (53.3)
≥20 yrs	5 (33.3)

**Table 2:** Results of all rounds of the Delphi study.

	**Questionnaire** **about the Need for Exercise**	**1st Round**			**2nd Round**			**Final Round**		
		**Median**	**IQR**	**% of** **Agreement**	**Median**	**IQR**	**% of** **Agreement**	**Median**	**IQR**	**% of** **Agreement**
1	Is exercise necessary to treat preexisting sarcopenia with hemiplegic stroke?	6.00	1.00	100.00	6.00	1.00	100.00	6.00	1.00	100.00
	**Questionnaire** **about Exercise Priorities**	**1st Round**			**2nd Round**			**Final Round**		
		**Median**	**IQR**	**% of** **Agreement**	**Median**	**IQR**	**% of** **Agreement**	**Median**	**IQR**	**% of** **Agreement**
Theme 1—General; Does exercise need to be										
1	Modified to each patient	6.00	2.00	93.33	6.00	1.50	100.00	6.00	1.00	100.00
2	Simple	6.00	0.50	93.33	6.00	0.50	93.33	6.00	0.50	100.00
3	Realistic	6.00	1.50	93.33	6.00	0.50	93.33	6.00	0.00	93.33
4	An achievable challenge	6.00	3.00	66.67	6.00	1.00	80.00	6.00	0.50	86.67
5	Acceptable to the patient	7.00	1.00	100.00	6.00	1.00	100.00	6.00	1.00	100.00
6	Appropriately time consuming	6.00	1.00	100.00	6.00	1.00	100.00	6.00	1.00	100.00
7	Adhered to by the patient	7.00	1.00	100.00	7.00	1.00	100.00	7.00	1.00	100.00
8	Decided in collaboration with the patient	5.00	1.50	73.33	5.00	1.00	80.00	5.00	1.00	93.33
9	Decided considering functional status and goals	7.00	1.00	100.00	7.00	0.50	100.00	7.00	0.00	100.00
10	Decided to affect neuromuscular performance	6.00	1.50	93.33	6.00	1.00	93.33	6.00	0.50	100.00
11	Decided to improve subjective symptoms	5.00	1.50	73.33	5.00	1.00	80.00	5.00	1.00	86.67
12	Monitored by the patient	6.00	1.50	80.00	6.00	0.50	100.00	6.00	0.00	100.00
13	Adapted to rehabilitation stage	6.00	1.00	100.00	6.00	1.00	100.00	6.00	0.50	100.00
14	Taught to the patient in advance	6.00	2.00	100.00	6.00	1.00	100.00	6.00	0.50	100.00
15	Decided considering equipment availability	6.00	0.00	100.00	6.00	0.00	100.00	6.00	0.00	100.00
Theme 2—Exercise dosage										
1	Frequency	6.00	1.00	100.00	6.00	1.00	100.00	6.00	1.00	100.00
2	Intensity of patient’s effort	6.00	0.50	93.33	6.00	1.00	100.00	6.00	0.00	100.00
3	Load of objective weight	6.00	1.00	93.33	6.00	1.00	100.00	6.00	1.00	100.00
4	Repetitions per set	6.00	1.00	100.00	6.00	0.50	100.00	6.00	0.00	100.00
5	Number of Sets	6.00	0.50	100.00	6.00	0.50	93.33	6.00	0.00	93.33
6	Exercise duration	6.00	1.00	100.00	6.00	1.00	100.00	6.00	0.50	100.00
Theme 3—Exercise type										
1	Exercise sequence (e.g., stretching→strengthening)	6.00	1.50	93.33	6.00	1.50	100.00	6.00	1.50	93.33
2	Exercise position (e.g., supine, prone, sitting)	6.00	0.50	93.33	6.00	0.00	100.00	6.00	0.00	100.00
3	Target muscle	6.00	1.00	100.00	6.00	1.00	100.00	6.00	1.00	100.00
4	Range of movement	6.00	1.00	93.33	6.00	0.00	100.00	6.00	0.00	100.00
5	Direction of resistance	6.00	0.50	93.33	6.00	0.00	100.00	6.00	0.00	100.00
6	Use of equipment	6.00	1.00	93.33	6.00	1.00	93.33	6.00	1.00	86.67
7	Use of weight equipment (e.g., ankle weights)	5.00	1.00	93.33	5.00	1.00	86.67	5.00	0.50	80.00
8	Use of elastic equipment (e.g., Thera band)	5.00	1.00	93.33	5.00	1.00	86.67	5.00	0.50	86.67
9	Use of balancing equipment (e.g., wall or cane)	6.00	1.00	80.00	6.00	1.00	86.67	6.00	1.00	80.00
Theme 4—Additional to extremity strengthening exercise										
1	Aerobic exercise	6.00	1.00	93.33	6.00	1.00	100.00	6.00	0.50	100.00
2	Stretching exercise	6.00	1.00	93.33	6.00	1.00	100.00	6.00	1.00	100.00
3	Flexibility exercise (e.g., yoga)	5.00	1.50	73.33	5.00	1.00	80.00	5.00	1.00	80.00
4	Core muscles exercise (e.g., pilates)	6.00	1.00	86.67	6.00	1.00	93.33	6.00	1.00	100.00
Theme 5—Evaluation of exercise										
1	Technique complexity	6.00	1.50	73.33	5.00	1.00	100.00	5.00	1.00	93.33
2	Patient effort	6.00	0.00	100.00	6.00	0.00	100.00	6.00	0.00	100.00
3	Patient compliance	6.00	0.50	100.00	6.00	0.50	100.00	6.00	0.00	100.00
4	Patient pain during exercise	6.00	1.00	100.00	6.00	0.50	100.00	6.00	0.00	100.00
5	Patient pain after exercise	7.00	1.00	100.00	6.00	1.00	100.00	6.00	0.50	100.00
6	Patient fatigue during exercise	6.00	1.00	100.00	6.00	0.50	100.00	6.00	0.00	100.00
7	Patient fatigue after exercise	6.00	1.00	100.00	6.00	1.00	100.00	6.00	0.00	100.00
Theme 6—Progressive overload										
1	Does the patient need progressive overload exercise?	7.00	1.00	93.33	7.00	1.00	100.00	7.00	1.00	100.00
Theme 7—When to overload										
1	When the patient no longer feels exercise is difficult	6.00	0.50	93.33	6.00	0.50	100.00	6.00	0.00	100.00
2	When the patient is not fatigued	6.00	0.00	93.33	6.00	0.00	100.00	6.00	0.00	100.00
3	When the patient feels ready	6.00	1.00	80.00	6.00	0.50	93.33	6.00	0.00	93.33
4	When the patient achieves functional goals	6.00	1.50	93.33	6.00	1.00	100.00	6.00	0.50	100.00
5	When the patient gains improvement of neuromuscular performance	6.00	0.50	86.67	6.00	0.00	93.33	6.00	0.00	100.00
6	When symptoms decrease	5.00	1.00	80.00	5.00	1.00	86.67	5.00	1.00	93.33
Theme 8—How to overload										
1	Considering patient’s functional activity	7.00	1.00	100.00	7.00	0.50	100.00	7.00	0.00	100.00
2	Frequency	6.00	1.00	93.33	6.00	0.50	100.00	6.00	0.50	100.00
3	Intensity of patient’s effort	6.00	1.50	93.33	6.00	1.00	100.00	6.00	1.00	100.00
4	Load of objective weight	6.00	1.00	93.33	6.00	0.00	100.00	6.00	0.00	100.00
5	Repetitions per set	6.00	1.00	93.33	6.00	0.50	93.33	6.00	0.00	93.33
6	Number of sets	6.00	0.50	93.33	6.00	1.00	86.67	6.00	0.50	93.33
7	Change of exercise position	6.00	1.00	93.33	6.00	1.00	100.00	6.00	0.50	100.00
8	Change of range of movement	6.00	1.00	100.00	6.00	0.50	100.00	6.00	0.00	100.00
9	Addition of exercise equipment	5.00	1.00	86.67	5.00	1.00	100.00	5.00	1.00	100.00

Descriptive statistics, such as median, IQR, and percentage of agreement were calculated for eight themes and 48 items. Items shaded in gray indicate those that did not reach consensus.

**Table 3:** Percentage of agreement and strong agreement among all rounds in the Delphi study.

	1st Round		2nd Round		Final Round	
	% of Agreement	% of Strong Agreement	% of Agreement	% of Strong Agreement	% of Agreement	% of Strong Agreement
Theme 1						
1	93.33	66.67	100.00	73.33	100.00	80.00
2	93.33	73.33	93.33	73.33	100.00	73.33
3	93.33	66.67	93.33	73.33	93.33	80.00
4	66.67	66.67	80.00	66.67	86.67	73.33
5	100.00	93.33	100.00	93.33	100.00	86.67
6	100.00	93.33	100.00	93.33	100.00	93.33
7	100.00	80.00	100.00	86.67	100.00	93.33
8	73.33	33.33	80.00	46.67	93.33	46.67
9	100.00	86.67	100.00	100.00	100.00	100.00
10	93.33	73.33	93.33	73.33	100.00	80.00
11	73.33	46.67	80.00	40.00	86.67	46.67
12	80.00	66.67	100.00	73.33	100.00	86.67
13	100.00	93.33	100.00	93.33	100.00	93.33
14	100.00	66.67	100.00	86.67	100.00	93.33
15	100.00	80.00	100.00	86.67	100.00	93.33
Theme 2						
1	100.00	93.33	100.00	100.00	100.00	100.00
2	93.33	80.00	100.00	73.33	100.00	80.00
3	93.33	66.67	100.00	66.67	100.00	66.67
4	100.00	80.00	100.00	86.67	100.00	86.67
5	100.00	73.33	93.33	73.33	93.33	80.00
6	100.00	73.33	100.00	73.33	100.00	73.33
Theme 3						
1	93.33	66.67	100.00	66.67	93.33	60.00
2	93.33	80.00	100.00	86.67	100.00	93.33
3	100.00	86.67	100.00	93.33	100.00	86.67
4	93.33	93.33	100.00	93.33	100.00	93.33
5	93.33	86.67	100.00	86.67	100.00	86.67
6	93.33	53.33	93.33	60.00	86.67	60.00
7	93.33	33.33	86.67	40.00	80.00	26.67
8	93.33	40.00	86.67	40.00	86.67	26.67
9	80.00	60.00	86.67	66.67	80.00	60.00
Theme 4						
1	93.33	60.00	100.00	66.67	100.00	73.33
2	93.33	80.00	100.00	93.33	100.00	86.67
3	73.33	40.00	80.00	40.00	80.00	33.33
4	86.67	60.00	93.33	66.67	100.00	66.67
Theme 5						
1	73.33	53.33	100.00	40.00	93.33	40.00
2	100.00	86.67	100.00	93.33	100.00	93.33
3	100.00	93.33	100.00	93.33	100.00	93.33
4	100.00	86.67	100.00	100.00	100.00	93.33
5	100.00	80.00	100.00	93.33	100.00	86.67
6	100.00	93.33	100.00	93.33	100.00	86.67
7	100.00	93.33	100.00	93.33	100.00	86.67
Theme 6						
1	93.33	93.33	100.00	100.00	100.00	93.33
Theme 7						
1	93.33	80.00	100.00	86.67	100.00	93.33
2	93.33	80.00	100.00	86.67	100.00	86.67
3	80.00	53.33	93.33	73.33	93.33	86.67
4	93.33	73.33	100.00	86.67	100.00	86.67
5	86.67	80.00	93.33	86.67	100.00	93.33
6	80.00	40.00	86.67	33.33	93.33	33.33
Theme 8						
1	100.00	80.00	100.00	100.00	100.00	100.00
2	93.33	53.33	100.00	73.33	100.00	80.00
3	93.33	66.67	100.00	80.00	100.00	80.00
4	93.33	66.67	100.00	80.00	100.00	80.00
5	93.33	80.00	93.33	86.67	93.33	93.33
6	93.33	73.33	86.67	66.67	93.33	73.33
7	93.33	60.00	100.00	66.67	100.00	73.33
8	100.00	60.00	100.00	73.33	100.00	86.67
9	86.67	40.00	100.00	40.00	100.00	33.33

The trend of the percentage of agreement and strong agreement in all rounds is presented. Items that did not reach consensus (<75%) are shaded in light gray. Items <50% of strong agreement are shaded in dark gray.

**Table 4:** Kendall’s coefficient of concordance for the Delphi study.

	Final Round				
	N	Kendall W	χ^2^	df	*p*
All items in all themes	15	0.319	268.094	56	<0.00 1*
Theme 1—General	15	0.303	63.679	14	<0.001 *
Theme 2—Exercise dosage	15	0.228	17.073	5	0.004 *
Theme 3—Exercise type	15	0.379	45.456	8	<0.001 *
Theme 4—Additional exercise	15	0.378	17.012	3	0.001 *
Theme 5—Evaluation of exercise	15	0.394	35.486	6	<0.001 *
Theme 6—Progressive overload	15	NA	NA	NA	NA
Theme 7—When to overload	15	0.311	23.296	5	<0.001 *
Theme 8—How to overload	15	0.391	46.860	8	<0.001 *

* Kendall’s coefficient of concordance was used to assess significant inter-expert agreement across items (*p* < 0.05). All Kendall’s W, the strength of inter-expert agreement, ranged from 0.2 to 0.4, indicating fair agreement. NA; Not Available.

**Table 5:** Standard and strong recommendations on the exercise priorities to treat preexisting sarcopenia with stroke hemiplegia.

Category	Priority
General	Modification/Simplicity/Reality/Achievable challenge/Acceptability/Proper time/Adherence/Collaboration with the patient/Functional status and goals of the patients/Effect on neuromuscular performance of the patient/Improvement of patient symptoms/Patient monitoring/Adaptation of rehabilitation stage/Education about exercise for the patient/Equipment availability
Dosage	Frequency/Intensity/Load/Repetitions per set/Number of sets/Exercise duration
Type	Sequence/Position/Target muscle/Range of movement/Direction of resistance/Use of equipment/Use of weight equipment/Use of elastic equipment/Use of balancing equipment
Additional exercise	Aerobic/Stretching/Flexibility/Core muscle exercise
Evaluation	Complexity/Effort/Compliance/Pain during exercise/after exercise/Fatigue during exercise/after exercise
Progressive overload	Need
When to overload	When the patient feels exercise is not difficult/When the patient is not fatigued/when the patient feels ready/When the patient achieves goals/When the patient gains improved neuromuscular performance/When the patient’s symptoms decrease
How to overload	Consideration of patient’s function/Frequency/Intensity/Load/Repetitions per set/Number of sets/Change position/Change range of movement/Additional equipment

The standard recommendation (57 items) was based on a consensus using the percentage of agreement, and the strong recommendation (49 items) was based on using the percentage of strong agreement. The strong recommendation was almost the same as the standard version except for the items shaded in gray.

## Data Availability

All data generated or analyzed during this study are included in this article and the Supplementary Materials. Further enquiries can be directed to the corresponding author.

## Supplementary Materials

The following supporting information can be downloaded at: https://www.mdpi.com/article/10.3390/life14030332/s1, Table S1: The details of all panelists in the Delphi study.
