# Supplementary material for: An Exercise Prescription for Patients with Stroke and Sarcopenia Based on the Modified Delphi Study
Source: Life (Basel). 2024 Mar 1;14(3):332. doi: 10.3390/life14030332 (PMC10971383; doi:10.3390/life14030332)
Supplement: Supplementary file 1 [file life-14-00332-s001.zip › life-2870086-supplementary.pdf]

Table S1. The details of all panelists in the Delphi study.

ID	Age	Sex*	Profession	Professional experience	Experience period (yrs)	Affiliation	Hospital infomation**
1	46	M	Medical doctor and Professor (Rehabilitation medicine)	✓ M.D.<br>✓ Medical specialist in Rehabilitatoin Medicine<br>✓ Professor and Former Director of Medical Affairs of National Traffic Injury Rehabilitation Hosipital-Seoul National University<br>✓ Academic Director, Korean Society of Rehabilitation Robot<br>✓ Member of Academic Committee of Korean Society for NeuroRehabilitation<br>✓ Member of Policy Committee of Korean Academy of Geriatric Rehabilitation Medicine	21	University hospital	General hopsital / 10 departments / About 250 patients
2	43	F	Medical doctor and Professor (Rehabilitation medicine)	✓ M.D.<br>✓ Medical specialist in Rehabilitatoin Medicine<br>✓ Member of Clinical Practice Guidelines Committee of Korean Academy of Rehabilitation Medicine<br>✓ Member of Education Committee of Korean Society for NeuroRehabilitation	15	University hospital	General hopsital / 10 departments / About 250 patients
3	42	F	Medical doctor and Professor (Rehabilitation medicine)	✓ M.D.<br>✓ Medical specialist in Rehabilitatoin Medicine<br>✓ Member of Research Planning Committee of Korean Academy of Rehabilitation Medicine	14	University hospital	General hopsital / 45 departments / About 1,400 patients
4	50	M	Medical doctor and Professor (Neurosurgery)	✓ M.D., Ph.D.<br>✓ Medical specialist in Neurosurgery<br>✓ Chief board member in General secretary of the Korean Society of Stereotactic and Functional Neurosurgery<br>✓ Editor of Journal of Korean Neurosurgical Society	26	University hospital	General hopsital / 38 departments / About 800 patients
5	48	M	Medical doctor and Professor (Neurology)	✓ M.D., Ph.D.<br>✓ Medical specialist in Neurology<br>✓ Member of the Korean Epilepsy Society<br>✓ Member of the Korean Neurological Association.<br>✓ Best Poster Awards in 17th World Congress on Controveries in Neurology in 2023	25	University hospital	General hopsital / 38 departments / About 800 patients
6	36	M	Physical therapist	✓ Registered therapist<br>✓ Team leader<br>✓ Organization manager of Korean Physical Therapy Association	14	University hospital	General hopsital / 38 departments / About 800 patients

ID	Age	Sex*	Profession	Professional experience	Experience period (yrs)	Affiliation	Hospital infomation**
7	36	M	Physical therapist	✓ Registered therapist<br>✓ Team leader<br>✓ Practice therapist instructor of the training course of central nervous system developmental rehabilitation by Korean Academy of Rehabilitation Medicine	11	University hospital	General hopsital / 38 departments / About 800 patients
8	33	M	Physical therapist	✓ Registered therapist<br>✓ Team leader	8	Rehabilitation hospital	Rehabilitation specialty hospital / 2 departments / About 250 patients
9	35	F	Occupational therapist	✓ Registered therapist<br>✓ Team leader	14	Rehabilitation hospital	Rehabilitation specialty hospital / 2 departments / About 250 patients
10	35	M	Occupational therapist	✓ Registered therapist<br>✓ Senior therapist	11	University hospital	General hopsital / 38 departments / About 800 patients
11	50	F	Nurse	✓ Registered nurse<br>✓ Head nurse	23	Rehabilitation hospital	Rehabilitation specialty hospital / 2 departments / About 250 patients
12	47	Fe	Nurse	✓ Registered nurse<br>✓ Head nurse	25	Rehabilitation hospital	Rehabilitation specialty hospital / 2 departments / About 250 patients
13	59	F	Nurse	✓ Registered nurse<br>✓ Head nurse	15	Rehabilitation hospital	Rehabilitation specialty hospital / 2 departments / About 250 patients
14	32	F	Nurse	✓ Registered nurse<br>✓ Team leader	11	University hospital	General hopsital / 38 departments / About 800 patients
15	28	F	Nurse	✓ Registered nurse<br>✓ Researcher in the rehabilitation field	5	University hospital	General hopsital / 38 departments / About 800 patients

* M means male and F means female in section of Sex. ** Hospital information includes type of hospital, number o f specialized medical departments, and number of hospitalized patients
